# Hospital ethics reflection groups: a learning and development resource for clinical practice

**DOI:** 10.1186/s12910-019-0415-5

**Published:** 2019-10-24

**Authors:** H. Bruun, L. Huniche, E. Stenager, C. B. Mogensen, R. Pedersen

**Affiliations:** 1 Focused Research Unit in Psychiatry, Institute of Regional Health Research, University of Southern Denmark, Odense, Denmark; 2 Department of Psychology, Faculty of Health Sciences, University of Southern Denmark, Odense, Denmark; 3 Focused Research Unit in Emergency Medicine, Institute for Regional Health Research, University of Southern Denmark, Odense, Denmark; 4 Center for Medical Ethics, Institute of Health and Society, University of Oslo, Oslo, Norway

**Keywords:** Ethics reflection groups, Action research, Evaluation, Significance, Psychiatric hospital, Emergency hospital

## Abstract

**Background:**

An ethics reflection group (ERG) is one of a number of ethics support services developed to better handle ethical challenges in healthcare. The aim of this article is to evaluate the significance of ERGs in psychiatric and general hospital departments in Denmark.

**Methods:**

This is a qualitative action research study, including systematic text condensation of 28 individual interviews and 4 focus groups with clinicians, ethics facilitators and ward managers. Short written descriptions of the ethical challenges presented in the ERGs also informed the analysis of significance.

**Results:**

A recurring ethical challenge for clinicians, in a total of 63 cases described and assessed in 3 ethical reflection groups, is to strike a balance between respect for patient autonomy, paternalistic responsibility, professional responsibilities and institutional values. Both in psychiatric and general hospital departments, the study participants report a positive impact of ERG, which can be divided into three categories: 1) Significance for patients, 2) Significance for clinicians, and 3) Significance for ward managers. In wards characterized by short-time patient admissions, the cases assessed were retrospective and the beneficiaries of improved dialogue mainly future patients rather than the patients discussed in the specific ethical challenge presented. In wards with longer admissions, the patients concerned also benefitted from the dialogue in the ERG.

**Conclusion:**

This study indicates a positive significance and impact of ERGs; constituting an interdisciplinary learning resource for clinicians, creating significance for themselves, the ward managers and the organization. By introducing specific examples, this study indicates that ERGs have significance for the patients discussed in the specific ethical challenge, but mostly indirectly through learning among clinicians and development of clinical practice. More research is needed to further investigate the impact of ERGs seen from the perspectives of patients and relatives.

## Background

Everyday clinical practice includes ethical considerations or challenges. An ethical challenge can be defined as a situation where there is doubt, uncertainty or disagreement about what is morally good or right [1]. Ethical challenges are prevalent in all parts of modern healthcare services. Important examples include difficult interactions with patients and relatives, uncertainty about respecting patient autonomy and difficulties in responding to the needs or outbursts of relatives. Other examples are unease over unsafe or unequal care, or uncertainty about who should have the power over care decisions [2]. In psychiatry, the same themes are described, but with the addition of the moral challenges caused by the use of coercion [3, 4]. Altogether, ethical challenges are complex situations where clinicians must balance medical knowledge, legal considerations and the sometimes conflicting values and interests of the parties involved. In a specific situation, a patient might suffer due to inappropriate decisions or reduced quality of treatment and care. In the long term, both patients and clinicians might suffer as a result of inappropriate decision-making processes and professional negligence, or poor handling of ethical challenges in daily clinical practice [5].

Various clinical ethics support services (CESS) have been established to help clinicians deal with ethical challenges. Important examples are clinical ethics committees (CEC) [6–8], ethics consultation [9], ethics reflection groups (ERG) [10] and moral case deliberation (MCD) [11]. ERG and MCD are very similar.

Some distinctive features of CESS are outlined below in Table 1. Some of the key differences are that ethics consultation and ERG/MCD tend to be more decentralized than CECs, which are often organized on a hospital level. A medical culture with little tradition for sharing difficult cases outside the wards can be a barrier for clinicians when presenting ethical challenges in CECs [12]. CECs also tend to have more focus on the participation of all involved parties, and therefore patients/relatives participate more frequently. In general, the reason for implementing CESS is to build competencies among clinicians by supporting them in their deliberations on ethical challenges; however, sometimes CECs are also used in policy development or to draft general ethical guidelines [6, 8]. By contrast, the focus in ERG/MCD is on the reflection process of clinicians more than on the decisions made or on finding solutions to a clinical problem [13]. Thus, the key reason for implementing ERG/MCD is to initiate a learning process and test the potential benefits, e.g. relief of moral distress among staff [14].

**Table 1:** Different ethics support services

	CEC	Ethics consultation	ERG/ MCD
How does it work?	A standing committee, deliberation on specific cases or more overall ethical questions asked by the healthcare organizations, clinicians and patients or relatives	An individual or a small team visiting a ward routinely or on request to deliberate on specific ethical challenges from everyday life experienced by clinicians	Collaborative interdisciplinary groups deliberating on specific ethical challenges from clinical practice experienced by clinicians
How is it organized?	Within a hospital trust or more hospitals in a region	Can be an ethics consultant employed by the hospital or a permanent body, e.g. CECs	Within one or across more hospital wards or clinical units
Who facilitates?	Ethicists or sometimes a clinician trained as a facilitator	Ethicists or sometimes a clinician trained as a facilitator	A clinician trained as an ethics facilitator
Who participates?	Physicians, nurses, ethicists, lawyers, clergy and community representatives, and sometimes patients/relatives	Clinicians in the ward or the clinical unit	Clinicians in the ward or the clinical unit

Until recently, approaches like MCD/ERG were most often used in community care [15, 16] and nursing homes [17]. With the exception of a few studies in psychiatry [18, 19], hospitals tend to use CECs and ethics consultation. To our knowledge, this is the first implementation study on ERG that includes both psychiatric and general medical hospital departments.

In their literature review, Haan et al. summarize the impact of moral case deliberation in a healthcare setting [13]. Dividing their findings into themes, they describe that MCD can lead to:

- *changes that are brought about on a personal and inter-professional level, with regard to professional’s feelings of relief, relatedness and confidence; understanding of the perspectives of colleagues, one’s own perspective and the moral issue at stake; and awareness of the moral dimension of one’s work and awareness of the importance of reflection*
- *changes that are brought about in caring for patients and families, with regard to profession-related changes and quality of patient care*
- *changes that are brought about on an organizational level*

Among others [20–22], Lillemoen et al. [15] introduce a learning perspective to account for the significance of ERG. They argue that the significance of ethical reflection is that “*employees seem to define ethics in accordance with double loop learning when they explain ethics as a reflection on practice. Unlike the automated actions that characterized the participants’ previous practice, they have discovered new aspects of their own practice, they ask questions they did not ask before, and they have become aware of new ways to deal with ethical challenges”* [15]. They discuss the positive findings from ERGs in community health services, stating that ERGs meet a need for professional development by way of dialogue, reflection, professionalism and relationship; elements that may not be offered the best conditions for growth in a healthcare system focusing on efficiency, target management and evidence. In a modern hospital organization, clinicians are faced with new challenges, e.g. new treatments, changing expectations and patients’ rights, which is why reflecting on treatment goals and means becomes increasingly important.

In this study, an ERG is defined as an interdisciplinary group of clinicians reflecting on a specific ethical challenge from everyday clinical life. Each ERG was organized and led by two ethics facilitators. Participation of ward managers was optional. Each ERG met twice a month for 45–60 min. A modification of the SME model (a deliberation model developed at the Center for Medical Ethics (“SME” in Norwegian) at the University of Oslo) [6, 15] was used [23].

### Aims of the research project and this paper

This paper is part of a research project whose overall aim is to foster systematic ethics reflection amongst clinicians in hospital settings by engaging them in ERGs as part of managing the ethical challenges encountered in clinical practice.

The research project was inspired by the work of a CEC in psychiatry in the Region of Southern Denmark [23]. Bearing in mind the challenges facing CECs, this study sees ERGs as a supplement to the services of the CEC, thus focusing on expanding structured ethics reflection into the wards and into everyday clinical practice. Along with evaluating the significance of ERGs seen from the perspectives of clinicians, ethics facilitators and ward managers, a sub-project has explored the barriers and promoters in the implementation of ERG in hospitals [24].

The aim of this paper has been to evaluate the significance of hospital ethical reflection groups. The research question was: What is the significance of ERGs in psychiatric and general hospital departments in Denmark from the perspectives of clinicians, ethics facilitators and ward managers?

## Methods

Evaluating the potential benefits of ERG is to evaluate the significance of a complex intervention. Action research is a systematic approach to the investigation and development of knowledge of complex human activities whilst simultaneously letting insights inform improvement of practice or social change [25]. Action research links the production of knowledge to the improvement of practice in a dynamic action research cycle. The cycle involves: problem identification, planning, action, and evaluation. Based on the evaluation and lessons learned, the cycle is repeated. Moreover, in an action research framework contextual and interactional matters are essential [26]. Being a research project with a dual purpose; both engaging clinicians in ERGs as part of managing ethical challenges encountered in clinical practice and developing new knowledge on implementation and the perceived significance of ERGs, the action research methodology was chosen as the most appropriate.

To represent different hospital organizational settings, three ERGs were established: One in an emergency department (Site I), and two in the psychiatric department: one in an inpatient ward (Site IIA) and another in an outpatient clinic (Site IIB). An extended description of the structure of the research project can be found in Appendix 1.

The aim of this study is to evaluate “how “and “why” ERGs are significant to clinicians. To answer these kind of questions, qualitative methods are suitable. Different qualitative research methods were chosen to generate different kinds of knowledge. By using participant observation, insight into what actually happened during ERGs was attained, whereas individual interviews and focus groups gave a retrospective assessment of the significance of the ERGs. In individual interviews, the significance of ERGs was seen from the perspectives of different clinicians, whereas the interaction between participants in focus groups triggered more expressive and responsive viewpoints, imparting new knowledge and adding another angle on the ERGs [27]. All participants received both written and verbal information about the project, before giving written informed consent.

### Data collection

#### Participant observations

Participant observation is a traditional ethnographic research method. The researcher takes part in other people’s daily living, generating research data by writing ethnographic field notes [28]. Participant observation can be conducted with varying degree of researcher participation [29]. During implementation of the ERGs, the first author was a participant observer in both the education and training of the ethics facilitator. Afterwards, descriptions, reflections and considerations were tape-recorded. After implementation the ERGs worked unassisted for about one year. During that project phase, the first author visited each ERGs three times to supervise and guide the ethics facilitators. Except for one (a technical error), all supervisions were tape-recorded. One implementation strategy was to form a project group consisting of the ward managers, all ethics facilitators and the first author contributing as a participant observer. In meetings, notes were taken and afterwards turned into written summaries. At the termination of the project, an “end-of-study workshop” was held by the project group, including also the leaders of the departments. Here preliminary results were presented and decisions were made whether to continue or end the ERGs. The end-of-study workshop was tape-recorded.

#### Individual interviews and focus groups

Interviews were carried out following a semi-structured interview guide (Appendix 2) focusing on three overriding issues: 1) What is daily practice in the department? What kind of ethics challenges are there? 2) How was the ERG implemented in your department? What was it like to facilitate/participate in the ERG? 3) What is the significance of the ERG on daily practice? The first author conducted the individual interviews, lasting an average of 49 min, varying between 31 and 65 min. Also, the first author moderated the focus groups, lasting an average of 43 min, varying between 36 and 52 min. All interviews were tape-recorded and transcribed verbatim. During transcription and additional processing of data, great care has been taken to anonymize persons and places. In the results section, the pronouns *he* or *she* are used at random.

#### Selection of informants for individual interviews and focus groups

Participants for the individual interviews were chosen strategically and with a focus on variety [30]. Participants expected to be able to give “thick descriptions” were chosen [27]. Table 2 shows the educational background and the type of engagement in the project of each participant individually interviewed.

**Table 2:** Participants in individual interviews

Role in ERG	Educational background						
	Physician	Nurse	Psychologist	Occupational therapist	Radiographer	Auxiliary nurse	In total
Local leader	1	4					5
Ethics facilitator		5	1				6
Participant	3 + 1 student	5 + 2 students	1	3	1	1	17
In total	5	16	2	3	1	1	28

Due to time restrictions, it was difficult to organize focus groups. Therefore the ward managers were involved in the organization. At some sites, already planned staff meetings were used. As physicians often did not participate in staff meetings, physicians were unfortunately absent in the focus groups. Table 3 describes the participants in the focus groups.

**Table 3:** Participants in focus groups

Focus group	Number of participants	Staff meetings	Educational background
Site I	5		5 Nurses
Site IIA	10	X	3 Auxiliary nurses, 6 nurses, 1 physiotherapists
Site IIB	17	X	3 Auxiliary nurses, 8 nurses, 2 psychologists, 3occupational therapists, 1 social workers
Ethics facilitators	4		3 Nurses, 1 psychologists

***Written case descriptions:*** In order for the participants to keep track of the processes involved in the SME model during the deliberation, a whiteboard with anonymized key information was used. At the end of the session, the ethics facilitators made a short anonymized written summary of the case presented and/or took a photo of the whiteboard used, to capture key information.

After the implementation phase the ERGs worked unassisted for about one year. In that project period the first author visited each ERGs three times. Except for one all these supervisions of the ERGs were tape recorded. The remaining meetings were not tape-recorded. There were multiple reasons for that decision. Firstly, when no researcher was present in the ERGs challenges might occur when gathering informed consent among clinicians participating. Secondly tape-recording might affect the deliberation process negatively. Thirdly the research team were lacking resources to analyse the data generated.

### Data analysis

Systematic text condensation has been used as the analytic strategy [31]. NVivo 11 was used to systemize the data during the examination. All individual interviews and focus groups were read by the first author. Some of the co-authors (RP and LH) participated in the analytic process by reading interviews and focus group transcripts discussing and validating the interpretation and later on the categories and sub-categories.

The analytic approach was abductive, involving moving back and forth between an inductive focus and a deductive focus. First, the inductive focus was applied: getting an overall impression of data, looking for similarities and differences between sites, and starting to make categories based on the overall themes in the transcripts [32]. The overall impression was that, regarding the perceived significance of the ERGs, there were no significant differences between the three sites. However, one exception was the number of retrospective and prospective cases presented in each ERG.

When illustrating and describing the significance of the ERGs, informants referred to specific cases; therefore the 63 short written descriptions of ethical challenges presented in the ERGs were included in the analysis. The content of the cases has been analyzed in relation to the significance reported in focus groups and individual interviews. Also, the analysis focused on the significance of the ERGs seen from different perspectives: the clinicians participating in the ERG, the ethics facilitators, and the ward managers. Although patients didn’t participate, neither in the ERGs nor in the research project, the focus on significance for patients were still in focus in the analysis.

Later in the analytic process, a deductive focus was applied to inform the analysis by theoretical concepts and relevant research literature on the outcome and significance of CESS [1, 8, 16, 18, 19, 21, 33] and learning theory [34, 35]. During dialogues with the co-authors, and informed by the research questions, the categories were processed and rearranged.

During the analytic process, four categories and several sub-categories emerged. They are described in Table 4. However, it is important to keep in mind that there are overlaps between the categories, as changes important to clinicians are also often important to patients or ward managers.

**Table 4:** Analytical process

Categories	Sub-categories
Ethical challenges assessed in the ERG	• Ethical challenges
Significance for patients	• Significance for the patients in question • Significance for other patients
Significance for clinicians	• ERGs as creative and shared learning and problem-solving for clinicians ° Management of ethical challenges before and after the implementation of ERGs ° The significance of interdisciplinary participation in ERGs • Development of clinical practice • Increased ethical awareness and competence ° Awareness of ethics when carrying out daily clinical practice ° Awareness of the influence of the hospital on the ethical challenges encountered • Prevention of privatization of ethical challenges
Significance for managers and the organization	• A leadership tool • Significance for the working environment ° Influence on clinicians’ relation to managers ° Influence on team-based cooperation

## Results

The result section is structured according to the categories and sub-categories presented in Table 4.

### Ethical challenges assessed in the ERGs

Table 5 gives examples of typical ethical challenges presented and assessed in the ERGs.

**Table 5:** Examples of ethical challenges discussed

Case	Site I
1	**A patient living in a nursing home.** An elderly patient, with a severe reduction in cognitive and functional capacity, was admitted on suspicion of a hemorrhage in the intestinal tract. The physician prescribed taking a blood sample, but the patient resisted and removed her arm – strongly and repeatedly. The nurse was in doubt if the patient had been informed about and agreed to the procedure – and to what extent she was supposed to try to convince the patient to accept the blood sample.
2	**The bricklayer continued to work.** A bricklayer was working at a big construction site. Some building materials fell from high above, hitting him on the head. Fortunately, he was wearing a helmet, and for a while he continued to work. But then he started getting a headache and some pain in his neck. When the ambulance took him to the emergency room, he was – in line with the clinical guidelines – placed on a spine board and told not to move. Suddenly the bricklayer needed the bathroom, but as he was at risk of having an unstable neck fracture, he was not permitted to leave the spine board. In order not to violate the guidelines, the nurse tried for a long time to persuade the bricklayer to urinate in a bottle, but he could not. The nurse was in doubt about the limits of her responsibility.
3	**Every day a new physician was responsible for the ward round.** As a consequence, a nurse experienced different assessments of her patient by the physicians; one day more intensive treatment was prescribed, the next day further treatment was regarded futile. Torn between loyalty to the physicians and solidary with the patient, the nurse was in doubt about how to act.
	Site IIA and IIB
4	**Reduction in antipsychotic medication?** A patient asked the nurse for a reduction in the dose of antipsychotic medication because the patient wished for some of the psychotic experiences to return. The nurse was in doubt whether it would be accepted by the physician to increase psychotic symptoms on the request of a patient, or if she had to try to persuade the patient to continue on a high dose, although she understood and respected the wish expressed by her patient.
5	**Continuation of treatment although cure is unlikely.** A clinician experienced organizational pressure to end treatment of a patient he had been treating for a long time. The patient was mentally ill, he was an alcoholic, and the patient was often suicidal. Adherence to treatment was poor. Although the patient was not cured, treatment prevented a deterioration, and the relation to the clinician offered alleviation and comfort. The clinician assessed their relation to be of utmost importance to the patient, and he feared the patient might slowly die if their relationship ended.
6	**The staff was frustrated by a patient constantly calling for assistance.** A patient was suffering from both mental illness and alcohol abuse. The patient was annoying – to both fellow patients and the staff. Therefore he was told to stay in his room. But then he started constantly calling for assistance by ringing the bell in his room. The staff was frustrated and tried to avoid the patient.
7	**Fluctuating patient wish for termination of treatment.** A patient disabled by severe personality disorder asked, with varying intensity and conviction, to end her treatment. The clinicians felt certain that ongoing treatment could help the patient. The patient was in doubt whether she wanted to continue or terminate treatment. At the same time, the clinician experienced pressure from the managers to end treatment because resources on the ward were already tight. Also, there was doubt whether termination or continuation of treatment would be in the best interest of the patient.
8	**Involvement of a vulnerable relative.** A psychotic very aggressive patient insisted that he would only take the prescribed medicine if it was administered to him by a close family member. The family member accepted to do so, but at the same time she witnessed aggressive actions done by the patient. The family member was vulnerable too, suffering from mental illness.
9	**Was it all right to transfer the patient?** A patient severely affected by mental illness needed specialized treatment in a hospital in a neighboring city. It was decided to go through with the transfer of the patient although the patient refused to cooperate and was transferred not sufficiently dressed.

**Fig. 1:**
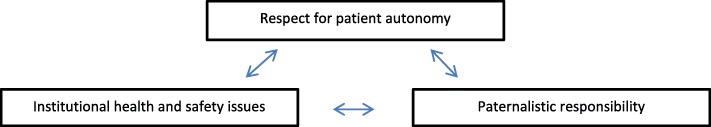
Different ethical concerns

In most cases, there were several conflicting concerns, and clinicians were struggling to find the best possible solution. An important element in all cases was respect for the patient’s autonomy. Generally, the cases revealed clinicians struggling to strike a balance between respect for patient autonomy, paternalistic responsibility, professional responsibilities, and institutional values – including efficient use of scarce resources. These different ethical concerns are described in Fig. 1.

These overarching conflicting concerns were seen in both psychiatric and somatic ERGs. However, the themes of the specific ethical challenges were different depending on the specific study site, the specific patient group and the clinical context. A frequent theme at Site I (emergency department) was “ethics at the end of life”. Also, as described in case no. 1, clinicians at Site I were often concerned about “too much treatment”. At the opposite end of the scale, as described in case no. 5, clinicians at Sites IIA and IIB (psychiatric departments) worried about the consequences of “too little treatment”.

Even though the majority of the cases came from psychiatric wards, there were only a few cases involving the use of formal coercion. On the other hand, there were several cases reflecting on informal use of influence, pressure, persuasion or power, and doubt about the limits of clinician’s responsibility, see cases no. 1, 2 and 4.

In some cases, different perspectives were represented by different professions. For example in case no. 1, the physician prescribed taking the blood sample according to guidelines, while the nurse alone experienced the patient rejecting to collaborate. In this case the physician was not necessarily aware of the ethical challenge experienced by the nurse.

None of the cases were presented by physicians.

### Significance for patients

#### Significance for the patients in question

At Site I, patients were admitted for hours only; therefore cases were only retrospective and it was mainly future patients that would benefit from the deliberation. By contrast, at Site IIB, patients were admitted for several months; therefore cases were often prospective, and the specific patient could often benefit from the deliberation.

Referring to the cases presented in Table 5, Table 6 gives examples of cases presented in which the patients concerned experienced changes in treatment plans as a result of deliberation in the ERG.

**Table 6:** Examples of significance for specific patients concerned by the ethical challenges assessed

Case	Significance
4	**Reduction in antipsychotic medication?** Contrary to expectation, the clinician experienced support from her colleagues – including the physician. As a consequence, she had a new unexpected possibility to meet the patient’s wish for a reduction in antipsychotic medicine.
5	**Continuation of treatment cure is unlikely.** Despite the absence of an obvious treatment effect, the clinician experienced her actions legitimized by her colleagues, and the patient’s treatment was continued.
6	**The staff was frustrated by a patient constantly calling for assistance.** The ERG gave room for changes in attitude towards the patient, describing him not as annoying but as suffering from loneliness. The stigmatization of banning him from the common room was addressed, and the actions of staff changed. As a consequence, the patient received more positive attention from staff.
7	**Fluctuating patient wish for termination of treatment.** The clinician worried about letting down a severely impaired patient if ending the treatment. It would be a violation of the duty to help, which was an important value to this clinician. During the deliberation process, respect for patient autonomy was introduced as a professional value. The result was a gradual, and much less dramatic, ending of treatment three months later, for the good of both the patient and the clinician.

#### Significance for other patients

There was agreement among clinicians that other patients benefitted from the ERG because the gaze of the clinicians on patients evolved. One stated that participation in ERGs increased the awareness that sometimes clinicians had thoughts and feelings that might be harmful to patient treatment**.** Some patients might, for example, trigger a sense of inadequacy or powerlessness in clinicians, and an impulse to end or withdraw from engagement when treating them.

*Some of the patients we have discussed, well, they aren’t always been that likeable and easy to help [ ] I think we’re going to be a bit more braver from now on, step back a bit and let the patient be in the driver’s seat, although you know it’s probably the road to nowhere, which can be difficult when you feel nothing happens at all [ ] Because some of our patients, they are not easy to help but we simply mustn’t let go of them.*

In the ERG, the patients and their specific stories were seen from a broader perspective. Compared to existing interdisciplinary fora, descriptions of patients were more comprehensive and held more nuances. One said that the descriptions of patients were “*fleshed out*”. Another said that the voices of the patients were more distinct and noticeable. That assessment in the ERG sometimes resulted in increased empathy and understanding of patients who were difficult to treat.

Another argument regarding patients in general benefitted from ERGs was the frequent concern for and deliberation on patient autonomy. Some said that their focus on patients’ right to self-determination had increased.

As a consequence, some described an increased focus on the differences between individual patients, e.g. in relation to the intensity of treatment when suffering from an incurable disease. Some clinicians said that their practice had changed a little; they listened more to patient perspectives, asked more questions and accepted to a greater extent the specific attitudes and opinions of the individual patient.

Although different values and attitudes were specifically asked for in the ERG, the outcome was often a more unified and homogeneous patient approach, caused by a deeper understanding of the patients and their situation. Some clinicians considered that a homogeneous approach gave better patient communication and prevented an unintended and potentially harmful effect of zigzagged decision-making.

### Significance for clinicians

#### ERGs as creative and shared learning and problem-solving for clinicians

##### Management of ethical challenges before and after the implementation of ERGs

Though most clinicians agreed that ethical challenges were a part of everyday clinical practice, they said that before implementation of the ERG, they often managed or dealt with these challenges in a random and unstructured way. As one clinician explained:

*A bit of informal chat in the office, and before you know it, since the two of us agree that’s the way to deal with it, that’s what we do around here. But then a few days later two other persons chat and share a few thoughts on something similar, and since they agree on the exact opposite, then that’s the way to handle things. It’s difficult to find a common approach.*

As the quote outlines, ethical challenges were sometimes discussed among a few colleagues. As a consequence of this unstructured assessment, the outcome tended to be random and inconsistent. Considerations and actions were not communicated among colleagues, and as a result they often faced the same challenges a few days later - sometimes concluding the complete opposite. Clinicians said that before the implementation of the ERG they had no organized forum for reflection on ethical challenges. When describing the significance of the ERGs, many clinicians compared the working conditions in the ERG with the work in existing interdisciplinary fora. Clinicians found the ERG different in several ways:

*Well, you could say it’s not something you bring up at the morning conference - well, the issues are there, of course, but we try to avoid talking about them, to be professional and focus on the purely medical aspects.*

The ERG was described as less hierarchical and judgmental. Some said that the ERG was a place for contemplation, for voicing doubt and asking the questions one might not dare to ask in other contexts. Also, in other fora, some felt the pressure of expectations and worried about saying something wrong. In other fora, the focus was strictly on treatment plans, and the perspective narrowly on medical issues. A physician acknowledged the description but explained that other clinicians, such as nurses, often asked for quick answers to complex problems, and that was one of the reasons why the working methods in other fora was more solution-oriented. By contrast, the ERG was described as a forum where different perspectives, opinions and ways of acting were encouraged. The atmosphere was creative, and the working methods required an open mind, allowing thoughts different from your own to come into play. There was room for curiosity, changing your opinion and for learning.

##### The significance of interdisciplinary participation in the ERG

There was general agreement on the positive contribution of interdisciplinary participation because it entailed different professional perspectives concerning scientific knowledge, legal responsibility, knowledge about the patient etc. The presence of different professions in the ERG increased the attention on the risk of being inflexible when assessing patient cases. One participant described himself as narrow-minded; another said that there was a risk of becoming “blinded by your own profession”. Some said that they were used to working in a world of their own and that there was a tendency to become more and more alike when working together in a team.

*And you’re sort of living in your own little bubble, and then you suddenly realize that, hey, the way things are done in your bubble is not the only way, that there are in fact other solutions*

For that reason, several clinicians evaluated the participation of other professions, other teams and departments as positive. Their own profession was put into perspective. Their professional blinkers were removed for a while. The presence of different healthcare professions made it possible to introduce new perspectives and solutions in relation to the cases assessed.

*You get to discuss the various scenarios from different angles. These scenarios, they can have more than one potential outcome.*

One said that some perspectives on a difficult case he himself had not thought of were presented to him by other clinicians in the ERG. He integrated the interpretation and understanding of the other clinicians into his own and used it the next time he found himself in a similar situation. He concluded that he had expanded his professional methods and practice.

Several clinicians said that the cases presented in the ERG were recognizable and concerned well-known ethical challenges. Although the deliberation concerned a specific case, the considerations and the ideas for action were highly relevant and usable in other and future situations.

#### Development of clinical practice

The ERGs influenced clinical practice in different ways. In some situations, the ERG caused specific changes among individual clinicians, staff or in overall ward practice.

As a consequence of deliberations on case no. 3 “Every day a new physician was responsible for the ward round” (Table 5), the nurse became more aware of the patients’ right to self-determination, and she changed her clinical practice when participating in ward rounds; before a round, she asked patients what they would like to ask about, and then she prepared the physician to talk about the subject mentioned by the patient.

A clinician included the reflection process of the ERG in her practice in a much more specific manner. Referring to the photo of the cues on the whiteboard or the short written summary of the ERG meetings, she explained how she had integrated the deliberation of the ERG into her work by using the saved photos as a kind of map showing the complexity of treatment situations. She said that the photos created an overview of a situation, which was useful if she got lost in everyday busy-ness. The cues also made sure that her line of thought and actions corresponded with the team’s. She said that the photo represented the best they could do in her team because all professions had been present at the ERG.

Yet another clinician said that for her one of the consequences of the deliberations in the ERG was that she had more courage to hold patients responsible for their conduct. She mentioned a patient threatening her when she was about to take a blood sample. She told the patient that he ought to know that having a blood sample taken hurts, and that it was no good threatening her because her increased nervousness could make the needle prick more painful. She also told him the blood sample was optional, and he was free to decline it. Shifting the responsibility was important to this clinician:

*Before I tended to take it personally, it became my problem, it all started and ended with me. But now I turn it upside down, so that it’s [ ] the patient’s problem, they have options, it up to them to choose what they want to do. [ ] It also means I’m not so sad any more when I go home. And it also means I can say, well, I did what I could, I did my best, with the resources we had available. So it’s easier for me, driving home, to say well that’s that, I can put it behind me, it was the patient’s own choice, or the choice of their relatives. It was not my choice [ ] Yes, it’s made me more aware that it’s OK that they make their own choices. And I can’t change them, or their choices, and I don’t have to.*

Moreover, deliberation in the ERG sometimes influenced more people, e.g. the staff or the overall practice of the ward. The significance of case no. 6 “The staff was frustrated by a patient constantly calling for assistance” (Table 5) was that the stigmatization of banning him from the common room was addressed, and the reactions of the staff towards the patient changed. Afterwards, staff became more accommodating towards the patient.

Also, the ERG made room for dialogue on issues otherwise ignored or overlooked, sometimes caused by lack of knowledge.

*It’s good that we take up these dilemmas, and that we find out what’s what, that it gets demystified, if you can put it like that, because it’s something, you know, coercion and stuff like that, it’s a bit taboo among us who work with somatic and not mental illness, it really is, so when coercion is brought up, people tend to sort of zone out a bit.*

As a result, the clinicians got new and important knowledge, e.g. concerning the use of coercion in the Danish Mental Health Services Act. Another example is case no. 2 “Bricklayer continued to work” (Table 5). The outcome of deliberating on the unintended consequences of strictly following the clinical guideline was that both the guideline and the clinical practice were looked into.

Reflection in the ERG on habits or practice on the ward sometimes led to organizational development. According to the Danish Mental Health Services Act, after an incidence of using coercion, clinicians must engage in a dialogue with the patient, in order to learn from the incident. The impact of deliberation on case no. 8 “Involvement of vulnerable relative” (Table 5) was that a new practice was introduced that widened the obligatory use of dialogue with patients after the use of coercion to also include dialogue with relatives involved in violent situations, in order to prevent coercion.

Every so often the outcome was less specific, more like a draft or an outline for potential actions. One clinician said that it was like getting some pieces of a puzzle which was not all done. But the pieces might have been sorted by colour, or maybe a part of the edge of the puzzle had been done. Another compared the ERG with a buffet from which you as a clinician could pick what you wanted depending on what you thought was usable.

### Increased ethical consciousness and competence

#### Awareness of ethics when carrying out daily clinical practice

It was reported that the ERG brought about a raised awareness of the ethical elements of clinical practice. The ERG gave an understanding of the “nagging feeling” clinicians might experience in some situations:

*You get this [] nagging feeling that this decision or choosing this direction, [ ] there’s something iffy about it, but it’s difficult to pinpoint exactly what. But once you’re in the context of [ERG] you realize that, yes, what was nagging you was actually ethical dilemmas, only they were sort of subconscious. [Afterwards] you see more clearly what it was that made you feel uncomfortable. [It’s] clarified things; it may still have been a rotten decision, or not such a good decision, but we had to make it. But now I know why I felt a bit queasy about it. So I suppose [ERG] can also bring clarification, instead of opening up a lot of new dilemmas.*

The daily decision-making process was seen in a normative context, and ethical considerations were understood as embedded in professional competence.

Concepts of theoretical ethics were “dusted off”. Some said it fostered a common language, which was important to the culture of the ward and the interaction with patients. One said that building up a common language on ethics was an important working tool in itself.

*I think it’s given us a common language. And I think that’s very important too, because when things get really hectic, as they do, then you have this common terminology you can just use without having to explain and explain.*

Some found themselves with a sharpened eye for the moral elements in everyday practice.

*I think that well, my eye for spotting ethical dilemmas has been sharpened. Before it was just something that, well, it was just a part of your life and job [ ] that did something to you, that got to you somehow. Now you spot immediately, yes, [ ] that’s an ethical dilemma. And then you can say to yourself, let’s give it a spin in the model [the SME model], let’s have a closer look at it [ ] there’s no need to think oh I don’t have the time to get to the bottom of this, let’s just move on.*

The ERG sharpened the ethical sensitivity of clinicians, and sometimes modifications of actions were made.

*But still you get these aha experiences, it’s amazing how many different opinions and attitudes people have, and how ethics is part of everything, even tiny things, like walking past a patient room without looking in [ ] and it makes you think ehmm perhaps I’ll do this differently next time, was it really OK to do so and so.*

#### Awareness of the influence of the hospital on the ethical challenges encountered

As described above (Indirect significance for patients) clinicians experienced increased attention on patients’ individuality and autonomy. Simultaneously it became more evident to some that the hospital setting did not always support that perspective. The daily clinical practice was described as busy, and finding time for reflection was therefore difficult. The everyday way of thinking was described as “black or white”. Some said that generally patients were quickly categorized and then treatment plans were drawn up.

The ERG offered a possibility to reflect on the ethics of the hospital as an organization creating an important frame of treatment. Some said that the ERG was the only forum in which the social and organizational context of hospitals was articulated. The everyday practice was seen from a bird’s eye view.

*Well, a lot hinges on packages these days, rapid assessment and treatment, triage, standards and guidelines and what not. And then they discover that a lot of [patients] don’t fit into any of the categories, and what are you supposed to do with them? There are so many requirements, so many targets and goals, and they often end up taking precedence over everything else.*

The above example illustrates the social context of treatment in hospitals, as well as the pressure put on clinicians to act in a specified way. As a consequence, some clinicians found their options for action restricted. How were they supposed to act when they had a patient falling “outside category”? Other clinicians described other kinds of organizational pressures: quick organizational changes and pressure to end treatment in consideration of scarce resources.

As a consequence of participation in the ERG, some clinicians described raised awareness of the organizational dimension of decision-making and a changed interpretation of the possibilities of action within the organization. After deliberation on case no. 4 “Reduction in antipsychotic medication?” described in Table 5, the clinician concluded:

*But it is actually possible to sort of nudge your way through the system, the system is not always as rigid and inflexible as we think. There are things that are possible, even if the system you work in seems quite restrictive.*

Another made the following conclusion about the conditions of treatment in the social context of the hospital:

*It [ERG] makes it legitimate to derogate from the rules if you can give good reasons for doing so. I think that’s something I’m more aware of now, it’s given people the courage to speak up and say I think this is the right thing to do, no matter what the standard says.*

### Prevention of privatization of ethical challenges

Before the implementation of the ERG, ethical challenges were frequently not assessed. At the same time, they were found to be difficult to handle and therefore often stored away. A physician said that he often suppressed those difficult questions. Some said that when they thought of work after working hours, they thought of troubles related to ethical challenges. One of the ward managers said that these kinds of questions were sometimes reflected on when clinicians were driving home after work with a colleague. Some also reflected with family members, but because few family members could relate to this kind of ethical challenges, many clinicians were left alone.

*It’s about the thoughts we all have, what we’re usually left to mull over on our own, here they’ve been said out loud and put into perspective [ ] … it’s been really good because it’s opened up this rag-bag of thoughts we’re all trying to make sense of. Now we’ve got some words to describe them, we’ve looked into what it’s really all about, and what other options there are.*

The ERG created a forum for bringing up issues usually coped with by clinicians individually, issues often hidden or suppressed in other interdisciplinary fora. The ERG offered a possibility to describe and share doubt and uncertainty in relation to decision-making and patient treatment which clinicians in their everyday busy lives experienced only as a stomach ache. The ERG made ethical challenges more visible. Moreover, the presence of the ERG legitimized that ethical challenges were talked about, whereas they were previously only mentioned in passing.

The ERG was described as an oasis or a retreat. The clinicians experienced that their colleagues faced the same ethical challenges, finding them as difficult as they did. Increased acceptance and understanding of ethical challenges surfaced as a challenging part of clinical practice. Some said that they came to realize that there was no correct result or answer. Others said that they had been affirmed; that they neither can nor are supposed to solve all problems like “little Tarzans”. There was an increased awareness and acceptance of the fact that sometimes all options are “bad options”.

An example is the case no. 9: “Was it right to transfer the patient?” presented in Table 5. Although not presented by the physician responsible, she participated in the ERG and reported that it had been significant to her because she was now more convinced that, among several poor ones, the option chosen was the least bad decision possible.

Clinicians described the support and affirmation of colleagues as important. They experienced that they were not alone when dealing with ethical challenges. They had a feeling of being in this together.

*Well, somehow it does affect you, if you’re thinking could I have acted differently, could I have handled this better, and you can’t help feeling reassured when you feel that the others understand you and support you and say that they’d probably have done the same. Even if, perhaps, what you did wasn’t the optimal solution.*

Some clinicians said that they unburdened themselves when sharing challenges, and that it was important to them both professionally and personally. Some reported that the ERG made it easier to cope with difficult ethical challenges.

*I felt much better afterwards. I felt so relieved, as if a huge weight had been lifted off my shoulders. Because I had felt so badly about it, I kept thinking of this patient and if she was OK.*

### Significance for ward managers and the organization

#### A leadership tool

The ward managers were aware that ethical challenges among clinicians might result in moral distress and burnout.

*Yes, I’m sure that’s what the staff do in their busy everyday life, they try to suppress these thoughts. Try to ignore them and then they develop stress symptoms, I think, simply because of this, you see, if you have to carry around such a baggage of unresolved issues.*

The ward managers also acknowledged that ethical challenges often drowned in the pressures of a hectic working schedule. Some said that clinicians might ask for quick solutions to difficult challenges. Sometimes ward managers felt that clinicians tried to escape responsibility. In these situations, the ERG was helpful. One manager said that the quality of the deliberation in the ERG on difficult cases was better than what he himself could provide. Therefore some ward managers referred clinicians to the ERG when they were approached by clinicians troubled by ethical challenges.

*I have to say I think it’s fantastic that I can refer them to it, that we have this opportunity. It think it’s a really positive thing [ ] I refer to it in many different situations. For instance when they [staff members] come by my office and tell me something, if the door’s open they often come in and say: phew, that was a difficult situation we had to handle yesterday, or have you heard about this or that episode? Then I say to them: what you tell me is so relevant and interesting I think it would be a great topic for discussion at our next meeting [ERG], and then I tell them when the next meeting is.*

The ward managers said that by participating in the ERG, they gained a different insight into the challenges experienced by clinicians – both organizational and personal. It was not unusual that the deliberation in the ERG caused them to take action in some way or other. An example was educational initiatives or training in specific procedures. Most ward managers were themselves experienced healthcare professionals, and they said that participation gave them valuable insight into the experiences of the young clinicians in the ward. At other times, they became aware of clinicians in need of special support. Also, the ethical challenges presented by experienced clinicians, whose work is usually performed under a large degree of self-management, were relevant to the ward managers. Most ward managers found the deliberation process relevant to their job. One said that she herself once presented an ethical challenge concerning a new initiative in the ward. She did that in order to get feedback on both good and unintended effects of the new clinical practice. The manager described that the atmosphere in the ERG was characterized by open-mindedness and mutual commitment to addressing and managing the ethical challenges.

*And I think that’s a really valuable thing. That we’re in this together, we agree this is a problem, so how do we solve it. There was this underlying willingness to not just talk about it but also do something about the issues discussed, that was my experience. [ ] It wasn’t about passing judgment or scoring points, although we talked about the optimal solution, which does imply a sort of value judgment, there was this open-mindedness, a willingness to think outside the box.*

Some ward managers said that it had been important to experience that clinicians really had a wish to participate and to feel their engagement in the reflection process. It made a deep impression, feeling the energy and engagement of the clinicians when participating in the ERG:

*There’s a special kind of energy in this. There really is. Even if we turn up feeling rather tired and we think, oh yeahhh, we’re going to do some reflecting now. But then we go into the room in the morning, and we leave with so much more energy. Something really happens in there.*

### Significance for the working environment

#### Influence on clinicians’ relation to ward managers

Although clinicians generally praised the ward managers for introducing the ERG, some reported that the ward managers’ participation in the ERG at times formed the deliberation process one-sidedly in line with the values or opinions held by the manager. Thus manager participation sometimes challenged openness and equality. Nevertheless, by introducing the ERG, clinicians found that their ward managers recognized the existence of difficult ethical questions in clinical practice. A clinician said that difficult decisions were respected in an entirely different manner when reflected upon than if the manager just told the clinician what to do.

*And then there’s respect for the ethical aspects of our job, at the end of the day that’s one of the reasons I think we like it … . it’s not always easy, is it?*

Some said they were taken more serious by the ward managers when they presented an ethical challenge recognizable to all as a group.

*And then the ward managers get to hear about these issues, and well ... then it’s not only you who says I’ve had this or that problem, and then they think, well, it’s probably just a one-off so there’s no need to act on it, but when they hear it from all of us, from different professional groups, then it’s something else.*

Some clinicians used the ERG as a forum for communication with the manager. Some mentioned that it was sometimes difficult to get to speak to the manager, as they were often busy or had their minds elsewhere. Clinicians described that in the ERGs the ward managers listened in a different manner. It was of great value that the ward managers attended a forum where there were time and calmness for deliberation.

#### Influence on team-based cooperation

The presence of the ERG affected the team-based cooperation positively. Deliberation on ethical challenges increased knowledge of individual clinicians and teams. Thoughts and attitudes causing a specific action were disclosed, causing a better understanding of why specific actions had been taken. This insight resulted in increased understanding between professionals, teams and departments. Prejudices were modified, and the work of others was respected in a more profound way.

*It’s really interesting to be part of it and especially when it a different team who presents their dilemma, because let’s face it [ ] we are sometimes inclined to [ ] agree among ourselves that the others’ jobs are so much easier than our own. And then it turns out that it isn’t.*

When reflecting together on ethical challenges, it was inevitable not to get to know each other on a more personal level.

*Well, if I’ve got a case and I tell them about it in the group, then I also tell a great deal about myself. And if I contribute in the discussion, then I also reveal some things about myself. And if I ask a question “Have you thought about ..?” then I also indicate what I think about it, and that’s also giving a little of yourself.*

Participating in the ERG, someone said, made him feel proud of working together with colleagues who had demonstrated that level of thoughtfulness and concern regarding the treatment of patients. Another said he was less reluctant to ask for assistance when in doubt or in need of counselling or support. When more personal values were shared openly among colleagues, they sensed that *we take good care of each other*.

## Discussion

As outlined in the introduction, the results of this research project are very much in line with the impact of MCD described by Haan et al. [13]. However, it also adds new knowledge. Illustrated by specific cases, this article describes the significance of ERG in three overall areas: significance for patients, significance for clinicians, significance for ward managers and the organization.

This study makes probable the impact that ERGs can have on patient treatment. However, in wards with short-time patient admissions only, the cases assessed were more often retrospective, and the beneficiaries of improved dialogue would be future patients rather than the patient in question in the specific ethical challenge. Other CESS methods, such as ethics consultation, establish the possibility for an ethics consultant to assist the team on request at short notice. On the other hand, the learning opportunity of ERGs has the potential of strengthening the long term confidence and capability of clinicians when handling everyday ethical challenges.

ERGs create a learning opportunity for clinicians which can lead to changes in clinical practices. Taking the previous lack of deliberation on ethical challenges in hospitals as the point of reference, clinicians described ERGs as less hierarchical and judgmental and with a more open and creative atmosphere. The fact that participation in the ERG was interdisciplinary entailed different professional perspectives concerning legal responsibility, knowledge about the patient etc. As a consequence, clinical practice was developed, causing specific changes among individual clinicians, staff and in overall ward practice.

Clinicians said that although the ERG did not always result in unequivocal solutions to ethical challenges, it offered an important possibility for sharing perspectives on ethical challenges, thus reducing the risk of privatizing such challenges and the decisions made to manage them. The ERGs was described as an oasis or a retreat. Some reported that the ERG made it easier to cope with ethical challenges. In spite of confusion around the terminology on ethical challenge, ethical dilemma and moral distress [5, 36], this study supports research literature indicating that reflection on ethical challenges contributes to reduction of moral distress [37, 38] described mostly among nurses, e.g. in intensive care units [39–41], but also among psychiatrists [42] and psychologists [43]. In this study clinicians point to ERGs as the only forum for discussing organizational limitations and nurse-physician interaction, described as important factors coursing moral distress [5, 44]. Moreover organizational conditions are widely recognized as part of what may cause moral distress; a situation when someone knows what action should be taken to protect a patient, but organizational constrains prohibit it [36]. However, Kälvemark et al. emphasize the importance of seeing moral distress not as an individual problem but as something to be addressed within the organization. They point it out when stating that *ethical judgments rarely refer to an individual person knowing certainly what is right or wrong. The process of ethical decision-making is much more complex. The reducing of moral distress is closely connected to the work organization and its provision of support structures for ethical discussions. These can help health care providers to live with the conflicts and ethical dilemmas that will always occur in their day-to-day practice* [44].

None of the physicians chose to present ethical challenges in the ERGs during this study. This may have been due to lack of time, but studies suggests that compared to other professions, physicians may to a lesser extent have an eye for the ethical challenges involved in their work [24]; also they may handle them differently [45, 46]. Another finding of this research project, described elsewhere [24], is that some physicians´ attitudes and interest in ethics reflection constitute a barrier to implementation of ERGs. *According to some physicians the job of a physician is described as opposed to engaging in a forum aspiring to be democratic, such as an ERG* [24]*.* As this insight emerged later on during the research project no initiatives were taken to engage physicians in the ERGs. More research is needed into the ethical challenges experienced by physicians, how they manage them, and if and how they may benefit from various types of CESS.

Ward managers acknowledged that ethical challenges often get pushed aside due to the pressure of everyday busy-ness. They also acknowledged the complexity of managing ethical challenges. Some ward managers stated that they referred clinicians to ERGs as a context for highly qualified ethical deliberation. Moreover, ward managers experienced that clinicians participated with more energy and engagement in reflections on handling ethical challenges.

The ERGs influenced the working environment. Team-based cooperation was strengthened, and collaboration between staff members and ward managers was reinforced. For some, the offer of participation in ERGs was seen as an expression of the ward managers’ responsiveness to the ethical challenges confronting clinicians in daily clinical practice. However, in some situations, manager participation tended to influence the deliberation in a direction that was formed by the opinion of the manager. As Weidema et al. note [33] it is important to emphasize that managers cannot expect ERGs to be a means to work towards a predefined goal because the outcome of deliberations depends on participants engagement in the open ended process. On the other hand, the findings in this project support Weidema et al. when they conclude that ERGs and MCDs can in fact contribute towards establishing dialogical interaction between clinicians and ward managers, resulting in a mutual encounter and further shared meaning-making in relation to ethical difficulties.

As described in the article “Implementing ethics reflection groups in hospitals: an action research study evaluating barriers and promotors” [24], different initiatives were taken based on the results of this study. In the emergency department after project termination the meetings in the ERG increased in frequency, from once every other week, to once a week. Also in the psychiatric department there was a wish to establish more ERGs. As described in the same paper barriers to the implementation were found to be: lack of time, administrative and financial management systems, and preconceptions among clinicians about ethics. In spite of positive results these barriers might complicate or even obstruct consolidation and dissemination of ERG in Danish hospitals.

The results of this research project show, that future ERG should initially be introduced at existing interdisciplinary fora – like staff meetings. In that way clinicians may slowly get to know the ERGs, the working method and the significance for themselves as clinicians, the ward managers and the organization, and the patients.

### Strengths and limitations

As this research project was carried out as a joint implementation of ERGs in both psychiatric and emergency departments, a variety of hospital organizational settings, staff members, types of patients and ethical challenges were represented. In this study, data was produced by using different research methods, such as participant observation, individual interviews, focus groups and written case descriptions. Moreover, preliminary research results were presented and validated in an “end-of-study workshop” at the termination of the project. Together, these elements increase the transferability of the study. However, researcher engagement in initiation, implementation and evaluation may have made the participants more reluctant to express criticism. To meet this challenge, critical participants and perspectives were actively requested, and during the individual interviews criticism specifically asked for and examined. An important limitation is that, although it has been demonstrated that patient participation in ERGs and CECs is possible and beneficial [47], patients were neither involved in the ERG nor in the evaluation. This decision was made on the basis of the additional challenges posed by involving patients and relatives in this study and at this stage considering the fact that CESS is a new initiative in Denmark. The decision was also influenced by the fact that only limited resources were available in the research team.

## Conclusion

This research project was conducted in a hospital setting, involving three departments in both psychiatric and general hospital departments. The ERGs were found to have had most significance among clinicians, with ERGs creating an interdisciplinary learning resource not found elsewhere in a Danish hospital setting. The deliberations in the ERG often led to changes in the way of acting among clinicians, staff in general and the overall practice of the ward. For clinicians, participation in ERG was also found to raise their awareness of ethics when carrying out daily clinical practice and of the influence that the hospital organization had on ethical challenges encountered in clinical practice. By helping to prevent ethical challenges from being privatized, this study makes it probable that ERG can help reduce moral distress among clinicians. ERGs were also found to have significance for ward managers and the hospital organization. Ward managers found ERGs relevant, e.g. as a leadership tool. As importantly, the ERG improved the working environment by improving team-based cooperation and the relation between clinicians and the ward managers. Significance for patients was found both for the patient concerned in the specific ethical challenge presented in ERGS, e.g. through changed treatment goals or changed approach to dealing with difficult patients, but mainly indirectly through the learning and development experienced by clinicians and potential changes in clinical practice. Involvement of patients as participants in ERGs and in research is a pressing need in order to further develop and better understand the value of ERG in a hospital setting. Involving patients as participants in ERGs and in research is much needed to further develop and better understand the value of ERG in hospital settings.

## Data Availability

In adherence to the regulations of the Danish Data Protection Agency and the period of time allowed for storage, data used and/or analyzed are available from the corresponding author on reasonable request.

## Appendix 1

### The structure of the research project

To represent different hospital organizational settings three ERGs were established: One in an emergency department (site I), and two in the psychiatric department: one in an inpatient ward (site IIA) and another in an outpatient clinic (site IIB).

**Description of participating departments**

**Table:**

	Site I	Site IIA	Site IIB
**Name**	Emergency department	Psychiatric inpatient ward	Psychiatric outpatient clinic
**Characterisation of patients**	Patients in need of urgent general or psychiatric medical attention	Patients experiencing deterioration in psychiatric disorder	Patients living at home, receiving specialized psychiatric assistance
**Subdivision of sites**	Two wards: 1. Quick assessment, patients stayed for hours 2. Patients stayed for few days	Patients were admitted for days or weeks	Three teams divided according to diagnosis, patients were admitted for several months
**Number of beds**	38	32	–

To implement the ERG different implementations strategies were used including courses, training and supervision of ethics facilitators. To support and form the implementation process a project group was formed, comprised of the first author, the head of each ward and all ethics facilitators. At the termination of the research project the project group conducted, together with the head of the departments, an “end-of-study workshop” evaluating preliminary results and experiences deciding on whether to continue the ERG or not.

## Appendix 2

### Interview guide

The interview guide consists of items to be covered and possible questions to be asked depending upon the person interviewed and his/her role in the ERG. In that way the interview guide was used in a flexible manner and it was slightly adjusted during the course of the study.

**Table:**

**Daily practice** • What is daily practice in the department? • What kind of ethical challenges are there? • How do you usually deal with ethical challenges in the department?	
**Implementation** • How was the ERG implemented in your department? • How did you participate in the implementation process? • What kind of barriers and promotors did you experience? • What are you doing in the ERG? • What is your experience of facilitating/participating in the ERG? • Try to describe the last time you participated in an ERG o Who participated? o What kind of problem or ethical challenge was assessed? o What was the outcome or result?	
**Significance** • What is an ERG to you, and how do you use it? • Do you remember an ethical challenge assessed in the ERG? Try to describe! • What is the significance – if any – of the ERG on daily clinical practice? o On collaboration with patients, relatives, colleagues, ward managers? o Can you give some examples? • What is the significance – if any – of the ERG on the department? • Have you experienced criticism of the ERG among colleagues? • Have you experienced that the ERG caused harm to anyone? • In what way does the ERG differ form/resemble other interdisciplinary fora? • How do you experience the presence of the ERG in daily practice in the department?	

## Abbreviations

CEC: Clinical ethics committee
CESS: Clinical ethics support service
ERG: Ethics reflection group
MCD: Moral case deliberation
SME: “Senter for Medisinsk Etikk” in Norwegian. In English: Center for Medical Ethics
